# Prolonged Intrahepatic Cholestasis After Acute Hepatitis E Infection: A Case Series and Genetic Analysis

**DOI:** 10.1111/jvh.70156

**Published:** 2026-03-09

**Authors:** Montserrat Fraga, Sophie Kasmi, Susanne N. Weber, Roman Liebe, Christine Sempoux, Ali Saadat, Jacques Fellay, Silke Kenngott‐Kelber, Vincent Zimmer, Frank Lammert, Marcin Krawczyk, Christoph Jüngst

**Affiliations:** ^1^ Division of Gastroenterology and Hepatology Lausanne University Hospital and University of Lausanne Lausanne Switzerland; ^2^ Department of Medicine II Saarland University Medical Center, Saarland University Homburg Germany; ^3^ Department of Gastroenterology, Hepatology and Transplant Medicine, Medical Faculty University of Duisburg‐Essen Essen Germany; ^4^ Institute of Pathology Lausanne University Hospital and University of Lausanne Lausanne Switzerland; ^5^ School of Life Sciences Ecole Polytechnique Fédérale de Lausanne Lausanne Switzerland; ^6^ Swiss Institute of Bioinformatics Lausanne Switzerland; ^7^ Abdominal Center Rüti Rüti Switzerland; ^8^ Department of Medicine Knappschaftsklinikum Saar Püttlingen Germany; ^9^ Center for Health Economics Research Hannover (CHERH) Hannover Medical School (MHH) Hannover Germany; ^10^ Department of Gastroenterology and Hepatology University Hospital Zurich Zurich Switzerland

**Keywords:** BRIC, cholestasis, HEV, human genetics, jaundice

## Abstract

Hepatitis E virus (HEV) is a leading cause of acute viral hepatitis worldwide. Whereas HEV infection is typically self‐limiting, rare cases of prolonged cholestasis have been reported. The underlying mechanisms remain unclear, though host genetic variation may contribute. This study aimed to investigate the role of genetic predisposition in HEV‐induced prolonged cholestasis by analysing variants in genes associated with hepatocanalicular transport. We performed a retrospective review of medical records from three university centres in Switzerland and Germany and identified five immunocompetent patients with prolonged cholestasis following acute HEV infections. Genetic analysis using next‐generation sequencing included a panel of five genes involved in cholestatic liver diseases (*ATP8B1, ABCB11*, *ABCB4, ABCC2* and *MYO5B*). Variant frequencies were evaluated using population reference databases and compared with a genetically characterised cohort of asymptomatic HEV‐infected blood donors. All five patients were male, with a median age of 59 years. The median duration of cholestasis exceeded 77 days. Two patients exhibited potentially pathogenic heterozygous variants: *ATP8B1* p.N45T in one patient and *MYO5B* p.K429R in another. Additionally, common *ABCB11* variants were detected in all patients, which might have contributed to cholestatic clinical presentation. In the asymptomatic HEV‐infected controls, the *MYO5B* p.K429R variant was absent, whereas the *ATP8B1* p.N45T variant was detected in only one individual in a heterozygous state. These case series illustrate that host genetics might influence the severity of HEV infection, particularly prolonged cholestatic jaundice. Further research is needed to explore the interaction between viral infections and host genetics in liver disorders.

## 1 Introduction

Hepatitis E virus (HEV), a small, non‐enveloped, positive‐sense single‐stranded RNA virus, is a leading cause of jaundice and hepatitis worldwide [1]. Approximately 950 million people, or about 12.5% of the global population, have been infected with HEV [2].

There are two distinct clinical and demographic scenarios. HEV genotypes 1 and 2, found in developing countries, infect only humans and spread via the faecal‐oral route. These genotypes cause hepatitis outbreaks in areas with poor sanitation and high mortality rates, particularly in pregnant women [1, 3]. In industrialised countries, pigs are the primary reservoir for HEV genotypes 3 and 4, and zoonotic transmission to humans typically occurs through the consumption of undercooked or raw meat [1, 3].

In immunocompetent individuals, acute autochthonous HEV infection is usually asymptomatic and self‐limiting [1, 3]. Extrahepatic manifestations mainly include neurological conditions, such as Guillain‐Barré and Parsonage‐Turner syndromes [1, 4]. In immunosuppressed individuals, chronic HEV infection may progress from chronic hepatitis to cirrhosis [1, 5]. Finally, rare cases of benign recurrent intrahepatic cholestasis (BRIC)‐like episodes with prolonged jaundice have been reported after acute HEV infection [6, 7, 8, 9].

The mechanisms underlying this broad range of clinical manifestations remain unclear, although host genetics is being increasingly discussed. A recent study [10] identified genetic variants related to the type I interferon response in patients with acute symptomatic and severe hepatitis after autochthonous HEV infection. Furthermore, two recent case reports suggested a genetic predisposition of hepatocanalicular transporter genes to developing a BRIC‐like episode after acute HEV infection [9, 11].

Therefore, we hypothesised that host genetic factors might play a role in influencing HEV‐induced prolonged cholestasis and explored the genetic contributions in otherwise healthy individuals who experienced prolonged cholestasis triggered by HEV.

## 2 Material and Methods

### 2.1 Study Population and Design

We conducted a retrospective review of the medical records of patients with prolonged cholestatic jaundice following a confirmed acute HEV infection at three university centres in Switzerland (Lausanne, Zürich) and Germany (Homburg). The electronic records and archives were systematically examined. Informed consent was obtained for all patients. The local ethics committee approved this study (Ärztekammer des Saarlandes, #271/11; CER‐VD protocol #2020–00197).

The inclusion criteria were [1] confirmed HEV infection, verified by positive HEV RNA in plasma and/or stool via polymerase chain reaction (PCR) testing, or by detection of anti‐HEV immunoglobulin M (anti‐HEV IgM); and [2] prolonged cholestatic jaundice following acute HEV infection, defined as total bilirubin levels exceeding 50 μmol/L for more than 30 days.

The exclusion criteria were as follows [1] patients with pre‐existing alcohol use disorder, chronic liver disease, advanced fibrosis/cirrhosis or immunosuppression [2] patients with alternative potential causes of cholestasis, including viral hepatitis other than HEV infection, autoimmune diseases, drugs or herbal supplements and biliary obstruction (systematically ruled out through abdominal ultrasound and/or magnetic resonance cholangiography).

### 2.2 Baseline Evaluation

The demographic data included age, sex and ethnicity. Clinical data encompassed relevant comorbidities, history of chronic liver disease, alcohol use disorder, ongoing treatments at presentation and any treatment for acute hepatitis E. Laboratory data included liver function tests, total bilirubin, serum albumin, coagulation parameters and creatinine levels. Virological data included anti‐HEV IgG and IgM serology, quantitative HEV RT‐PCR in plasma and/or stool samples. Pathological data included expert liver biopsy assessments when available. Genetic data consisted of next‐generation sequencing (NGS) for five genes associated with cholestatic liver conditions.

### 2.3 Next Generation‐Sequencing and Bioinformatic Analysis

Genomic DNA from peripheral EDTA blood was isolated using standard procedures (QiAamp DNA extraction; Qiagen, Hilden Germany). Library preparation for NGS was performed using Illumina DNA prep with enrichment (Illumina, San Diego, USA). Primers for the distinct genes (*ATP8B1*, *ABCB11*, *ABCB4*, *ABCC2* and *MYO5B*) were designed as custom panels in cooperation with Illumina.

Direct paired‐end NGS sequencing (2 × 150 bp) was performed on the Illumina MiniSeq platform, and the results were analysed using JSI SeqNext software (JSI, Ettenheim, Germany) at the Saarland University Medical Center, Germany. In patient 5 paired‐end NGS sequencing of solely *ATP8B1* and *ABCB11* was performed as part of the routine diagnostic procedure by the Institute of Medical Genetics at the University of Zurich, Switzerland. To gain further insight into the potential importance of the detected variants, we assessed their frequencies in the Genome Aggregation Database (gnomAD) (gnomad.broadinstitute.org).

### 2.4 Comparison Cohort

As a comparison cohort, we included asymptomatic HEV‐infected blood donors from a previously published Swiss multicentre genomic study [10]. Asymptomatic blood donors were identified through routine nationwide blood donation screening performed in Switzerland between January 2021 and October 2022, during which systematic HEV testing was conducted in accordance with national blood safety regulations. Acute HEV infection was confirmed by quantitative polymerase chain reaction (PCR). All individuals were adults (≥ 18 years) and underwent standardised clinical assessment using a structured questionnaire to ensure the absence of symptoms related to HEV infection. Only strictly asymptomatic donors were included. All these asymptomatic blood donors included in the comparison cohort had undergone whole‐genome sequencing (WGS) as part of the previously published Swiss multicentre genomic study.

## 3 Results

### 3.1 Study Participants

Five patients with acute hepatitis E‐induced prolonged cholestasis between May 2013 and February 2022 were retrospectively identified. Notably, patient 1 was already reported [9]. Extensive diagnostic workups, including imaging, ruled out biliary obstruction and alternative causes of jaundice.

**FIGURE 1:**
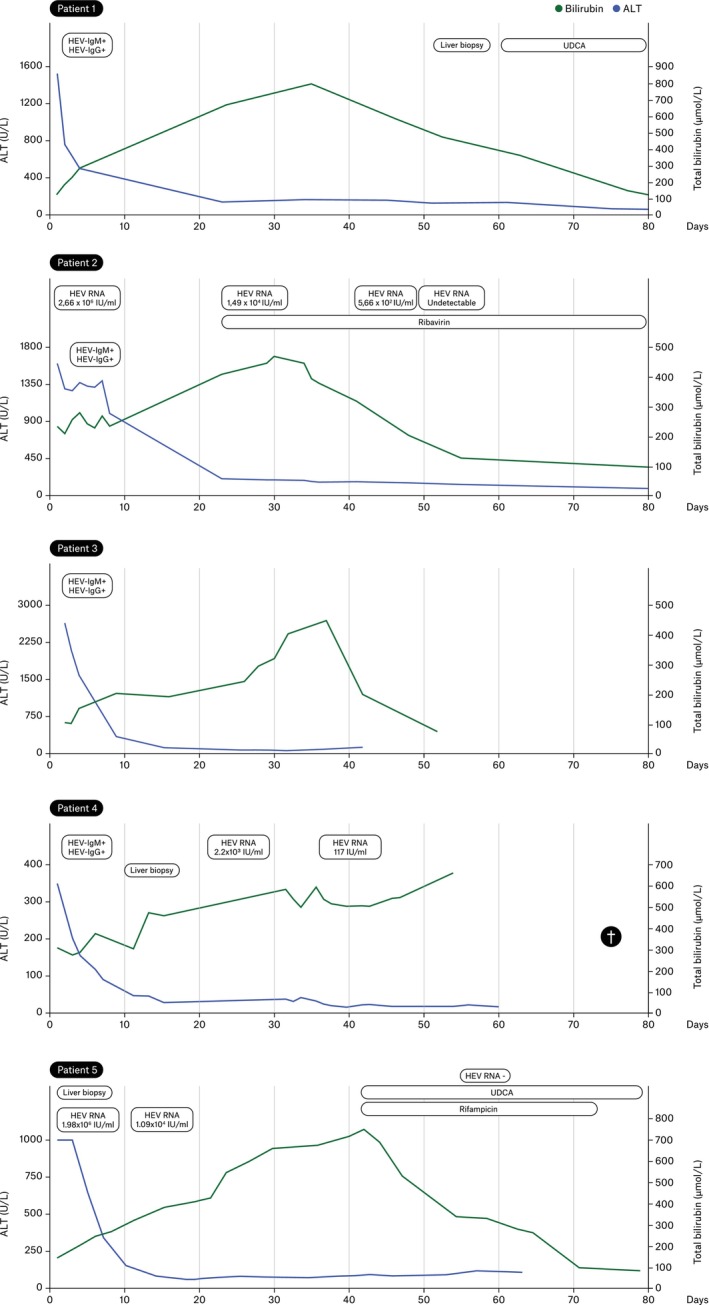
Clinical course of the five identified patients. UDCA, ursodeoxycholic acid; †, death.

All patients were men and of Caucasian origin with a median age of 59 years (range 36–83 years). Table 1 summarises the patients' characteristics and Figure 1 illustrates the clinical course of the five patients.

**TABLE 1:** Demographic, clinical, laboratory and pathological characteristics of the patients.

Patient	1	2	3	4	5
Age (years)	59	72	36	83	50
Sex	Male	Male	Male	Male	Male
Ethnicity	Caucasian	Caucasian	Caucasian	Caucasian	Caucasian
Serology (IgM/IgG)	IgM+/IgG+	IgM+/IgG+	IgM+/IgG+	IgM+/IgG+	IgM+/IgG+
HEV RNA (IU/ml)	Negative (d44 of jaundice)	2.6 × 10^6^	Negative (d28 of jaundice)	2.2 × 10^3^	1.9 × 10^6^
ALT peak (U/L)	1514	1606	2646	359	> 1000
ALP peak (U/L)	NA	208	157	188	304
Total bilirubin peak (μmol/L)	795	469	452	578	736
GGT peak (U/L)	NA	433	205	320	389
Jaundice duration (days)	83	88	> 52	> 60	> 77
Histology	Canalicular cholestasis, no hepatitis, no cholangitis	—	—	Severe cholestatic hepatitis	Canalicular cholestasis
Treatment	UDCA	Ribavirin	—	—	UDCA, Rifampicin
Clinical outcome	Cholestasis resolution	Cholestasis resolution	Cholestasis resolution	Death	Cholestasis resolution

*Note:* Jaundice was defined as total bilirubin superior to 50 μmol/L.

Abbreviations: ALP, Alkaline phosphatase; ALT, Alanine aminotransferase; GGT, gamma‐glutamyltransferase; NA, non‐available; UDCA, ursodeoxycholic acid.

The median alkaline phosphatase peak value was 198 U/L (range 157–304 U/L). The median gamma‐glutamyltranspeptidase (GGT) peak activity was 354 U/L (range 205–433 U/L). In all five patients, the GGT levels were low during the prolonged cholestasis. The median peak total bilirubin concentration was 578 μmol/L (range 452–795 μmol/L). The median duration of total bilirubin levels > 50 μmol/L was 77 days (range 52–88 days). In patient 4, serum bile acids measured on day 32 after presentation were elevated at 56 μmol/L (reference value ≤ 10 μmol/L).

**FIGURE 2:**
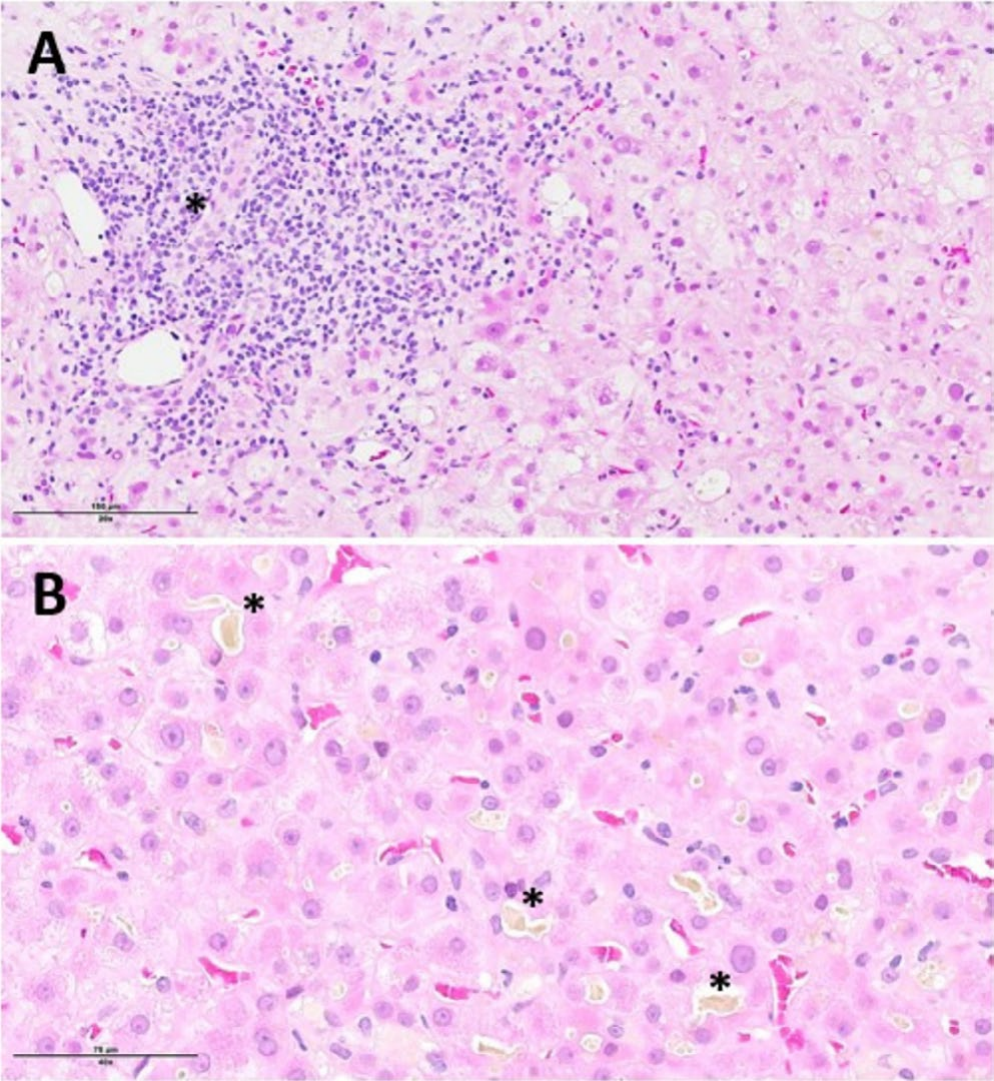
Results of liver biopsy in patient 4. A. Portal tracts: Diffuse inflammatory infiltration, mostly lymphocytic, with florid interface activity and no bile duct lesion (*) or significant ductular reaction. B. Lobules: Numerous bile thrombi in the canaliculi (*).

Three patients (patients 1, 4 and 5) underwent a liver biopsy. In patients 1 and 5, the biopsy showed canalicular cholestasis without signs of hepatitis or chronic cholangitis. A liver biopsy of patient 4 was performed 10 days after his initial presentation, demonstrating features of cholestatic hepatitis without fibrosis (Figure 2). One patient was treated with ursodeoxycholic acid (UDCA), and one patient was treated with ribavirin for 4 weeks until HEV RNA was undetectable in the blood and stool. A third patient was initially treated with UDCA without any impact on bilirubin levels and itch, and after additional treatment with rifampicin (150 mg/day), there was a prompt continuous improvement in bilirubin levels and itching. Two patients did not receive any specific drug treatment. Complete resolution of cholestasis was observed in four patients. Patient 4, an 83‐year‐old male, developed intractable pruritus with an unfavourable course of cholestasis. The patient's condition was further complicated in the late disease course by bacterial pneumonia, leading to death.

### 3.2 Comparison Cohort

The comparison cohort consisted of 16 asymptomatic blood donors with confirmed acute HEV infection [10]. Twelve of the 16 participants (75.0%) were male, with a median age of 56 years (range, 24–74 years). All individuals were of Caucasian origin. None had a history of chronic liver disease, and none were receiving immunosuppressive therapy at the time of diagnosis. No participant reported recent travel to regions endemic for HEV genotypes 1 or 2, supporting autochthonous HEV infection consistent with genotype 3 circulation in Switzerland. All individuals were asymptomatic at the time of HEV diagnosis, as documented by a standardised questionnaire specifically designed to exclude clinical manifestations of HEV infection. Quantitative HEV RNA PCR was performed in all participants, with a median viral load of 3.1 log_10_ IU/mL (range, 1.1 – 5.3 log_10_ IU/mL).

### 3.3 Genetic Analysis

Genetic analysis revealed multiple variants in the five genes analysed (Table 2). Based on gnomAD frequency (AF) in Europeans (non‐Finnish) and previously published findings, the following variants, observed in a heterozygous state, were considered potentially pathogenic: the rare missense variant *ATP8B1* p.N45T (gnomAD AF = 0.0055), which was detected in patient 1 [12, 13], and the ultra‐rare variant *MYO5B* p.K429R (gnomAD AF = 0.0000017), which was observed in patient 2.

**TABLE 2:** Results of NGS of *ATP8B1*, *ABCB11*, *ABCB4*, *MYO5B* and *ABCC2* genes. Potentially pathogenic variants are presented in bold.

Variant	Location and nucleotide alteration	Predicted effect	P1	P2	P3	P4	P5
**ATP8B1 variant**							
rs146599962	**c.134A > C**	**p.N45T**	Heterozygous				
rs319438	c. 696 T > C	p.D232D	Homozygous	Homozygous	Homozygous	Homozygous	Homozygous
rs319439	c.698 + 20C > T						Heterozygous
rs319443	c.811A > C	p.R271R	Homozygous	Homozygous	Homozygous	Homozygous	Homozygous
	c.2098‐8C > A		Heterozygous	Heterozygous	Heterozygous	Heterozygous	
	c.2098‐4C > A		Heterozygous	Heterozygous	Heterozygous	Heterozygous	
rs12968116	c.2855G > A	p.R952Q					Heterozygous
rs222581	c.3454G > A		Homozygous	Homozygous	Homozygous	Homozygous	
**ABCB11**							
**variant**							
rs4148776	c.99‐18 T > C		Heterozygous				
rs11568363	c.389 + 8G > A		Heterozygous				
	c.909–17,909–15		Heterozygous				
rs2287618	c.909–15A > G		Heterozygous	Heterozygous			Heterozygous
rs7563233	c.957A > G	p.G319G	Heterozygous				
rs2287622	c.1331C > T	p.A444V	Heterozygous	Heterozygous			Heterozygous
rs11568364	c.2029A > G	p.M677V	Heterozygous				
rs853772	c.2179‐17C > A		Homozygous	Heterozygous	Homozygous	Heterozygous	Homozygous
rs853789	c.2344‐17 T > C		Homozygous	Heterozygous	Homozygous	Heterozygous	Homozygous
rs497692	c.3084A > G	p.A1028A	Homozygous	Heterozygous	Homozygous	Heterozygous	Homozygous
	c.3882G > T	p.V1294V		Heterozygous			
**ABCB4 variant**							NA
rs2302387	c.175C > T		Heterozygous	Heterozygous		Heterozygous	
rs1202283	c.504C > T	p.L59L	Heterozygous	Heterozygous	Heterozygous	Homozygous	
rs2109505	c.711A > T	p.N168N	Heterozygous	Heterozygous			
rs2230028	c.1954A > G	p.I237I				Heterozygous	
rs31668	c.2211 + 16C > T	p.R652G	Heterozygous	Homozygous	Homozygous	Heterozygous	
rs31653	c.3508‐16 T > C		Heterozygous	Heterozygous	Homozygous	Heterozygous	
**MYO5B variant**							NA
rs1815930	c.376A > G	p.T126A	Homozygous	Homozygous	Homozygous	Homozygous	
rs17659179	c.921G > T	p.K307K				Heterozygous	
	**c.1286A > G**	**p.K429R**		Heterozygous			
rs17715416	c.1545 + 11 T > C				Homozygous		
	c.1586A > G			Heterozygous			
rs2298628	c.2049G > A	p.V683V	Heterozygous	Heterozygous	Heterozygous	Heterozygous	
rs2298624	c.2735G > A	p.R919H	Heterozygous			Heterozygous	
rs200219597	c.3163_3165dup	p.L1056dup	Heterozygous		Homozygous	Heterozygous	
rs2276176	c.3276 + 11 T > C		Heterozygous			Heterozygous	
rs3826579	c.3591C > T	p.Y1197Y	Heterozygous			Heterozygous	
rs488890	c.4315 + 5G > C			Homozygous		Heterozygous	
rs112417235	c.5062A > G	p.M1688V	Heterozygous				
**ABCC2 variant**							NA
rs927344	c.116A > T	p.Y39F	Homozygous	Homozygous	Homozygous	Homozygous	
rs17222561	c.1483A > G	p.K495E				Heterozygous	
rs3740066	c.3972C > T	p.I1324I		Heterozygous	Homozygous		

All other variants were considered benign or likely benign, although several have been described as minor contributors towards impaired bile secretion [14, 15].

WGS was performed in all 16 asymptomatic HEV‐infected blood donors as part of a previously published original research study [10]. Targeted analysis of the two variants of interest revealed that the *MYO5B* p.K429R variant was absent in all asymptomatic HEV‐infected blood donors, whereas the *ATP8B1* p.N45T variant was detected in only one control individual and exclusively in the heterozygous state.

## 4 Discussion

Although acute HEV infection is typically asymptomatic and self‐limiting, it can occasionally result in severe hepatitis and jaundice. It has been shown that host genetics may influence the clinical presentation of HEV infections [10]. Based on this background, we hypothesised that functional variants within hepatocanalicular bile salts or phospholipid transporters might contribute to severe and prolonged forms of HEV‐induced cholestasis [9].

Homozygous functional variants of genes involved in biliary lipid transport may cause severe forms of hereditary cholestasis, collectively termed progressive familial intrahepatic cholestasis (PFIC). Heterozygous variants of *ATP8B1* and *ABCB11* have been linked to intermittent prolonged cholestatic episodes, also known as benign recurrent intrahepatic cholestasis (BRIC) types 1 and 2, respectively [16]. Cholestasis with jaundice typically lasts several weeks to months in patients with BRIC, and GGT levels are typically low. Various trigger factors have been described such as drugs, hormones, surgery and viral infections especially in the upper respiratory tract [17]. Cholestatic phenotypes arise from an interplay between genetic predisposition and environmental factors, whereby carriers of risk variants exhibit cholestasis only when additional triggers compromise transporter expression or function, while remaining phenotypically unaffected in their absence.

We analysed five patients who presented with prolonged icteric cholestasis following acute HEV infection to investigate potential variants in genes associated with hepatocellular cholestasis. As a comparison cohort, we included asymptomatic blood donors with documented acute HEV infection who had undergone WGS in a previous study.

Genetic analysis of patients with HEV‐induced cholestatic hepatitis revealed a complex picture of multiple variants (Table 1), two of which deserve further attention.

To place our genetic findings in an appropriate population context, we assessed the frequency of all detected variants using the Genome Aggregation Database (gnomAD) to distinguish common background variation from potentially relevant rare alleles. Two heterozygous variants were retained based on their low population frequency and prior published data: *ATP8B1* p.N45T, a rare missense variant (gnomAD European non‐Finnish AF = 0.0055), and *MYO5B* p.K429R, an ultra‐rare variant (gnomAD AF = 0.0000017). These frequencies indeed indicate that both variants are uncommon in the general population, with *MYO5B* p.K429R being particularly rare.

In patient 1, we identified the heterozygous variant *ATP8B1* c.134A > C, resulting in an amino acid substitution of asparagine with threonine (p.N45T). *ATP8B1* acts as a flippase at the hepatocanalicular membrane and translocates phospholipids from the external to the cytoplasmic membrane leaflet and thereby maintains the canalicular membrane asymmetry. A disrupted membrane symmetry leads to impaired canalicular protein function, including the bile salt export pump (BESP), which is encoded by the *ABCB11* gene and represents the primary transporter of bile salts from the hepatocyte into bile, thereby contributing to cholestasis [18, 19]. This *ATP8B1* variant was initially described in a woman with intrahepatic cholestasis of pregnancy (ICP) [13]. Dixon et al. [12] identified six patients carrying the *ATP8B1* p.N45T mutation in a larger ICP cohort, in which the variant was observed at approximately twice the frequency reported in the general population. Importantly, the global minor allele frequency of this variant in gnomAD population reference databases is low and approximately 0.5%. This variant was also identified in the heterozygous form in a patient with BRIC [20].

In a second patient, we detected an ultra‐rare heterozygous variant in the *MYO5B* gene, resulting in the substitution of lysine with arginine (p.K429R). Notably, the global population prevalence of this variant is extremely low, estimated at 0.00017% in the gnomAD reference dataset. *MYO5B* encodes a protein involved in cell polarisation and the intracellular trafficking of transport proteins, such as BSEP to the canalicular membrane. Severe *MYO5B* mutations result in microvillus inclusion disease, a pronounced intestinal phenotype, whereas isolated cholestatic liver diseases such as chronic low GGT liver disease or recurrent episodes of cholestasis have also been reported [18, 21, 22]. The *MYO5B* variant p.K429R is very rare according to gnomAD (present in only two out of 1,180,010 alleles). The variant was localised in a highly conserved region of the gene, suggesting that it is functionally relevant. Furthermore, it has been shown that liver phenotypes result from relatively mild *MYO5B* variants rather than biallelic nonsense or frameshift mutations and can also occur in heterozygous carriers [22].

The rarity of the two identified variants supports a genetic susceptibility model for prolonged cholestatic hepatitis following acute HEV infection. Consistent with this, comparison with a WGS‐characterised asymptomatic HEV‐infected blood donor cohort showed absence of the ultra‐rare *MYO5B* p.K429R variant and only a single heterozygous carrier of the *ATP8B1* p.N45T variant. Despite limited sample size, these findings suggest that such variants are uncommon in uncomplicated HEV infection and may contribute to susceptibility to prolonged cholestasis.

Interestingly, a comparable susceptibility framework has been described in cholestatic drug‐induced liver injury (DILI). Although the specific variants identified in our cohort (*ATP8B1* p.N45T and *MYO5B* p.K429R) have not been reported in DILI, variants in other cholestasis‐related genes from our panel (particularly *ABCB11* and *ABCB4*) have been linked to cholestatic DILI. These observations suggest overlap at the gene and pathway level between cholestatic DILI and HEV‐associated cholestasis, despite differences at the individual variant level [23, 24].

Overall, we consider that both variants, *ATP8B1* p.N45T and *MYO5B* p.K429R, might have contributed to the observed phenotype, with the limitation that functional analyses are lacking. Several of the common variants detected in the *ABCB11* gene might also add to predisposition to cholestasis in these patients. The *ABCB11* c.3084A > G synonymous variant was found in all patients, either in the heterozygous or homozygous states. This risk variant has been linked to the development of preoperative jaundice and decreased protein and mRNA expression in patients with intrahepatic gallstones [15]. The *ABCB11* c.909–15A > G variant has been associated with a reduced response to ursodeoxycholic acid in patients with primary biliary cholangitis and was observed in three of the patients in our study [14].

In conclusion, NGS analysis of the total coding regions for the three most frequent PFIC‐associated genes *ATP8B1*, *ABCB11* and *ABCB4* (PFIC1‐3), as well as *MYO5B* (PFIC6) and *ABCC2* revealed potentially pathogenic variants in two patients, and several predisposing common *ABCB11* variants in all patients. The causative genes for the very rare subtypes PFIC4 (*TJP2*) and PFIC5 (*NR1H4*) were not analysed.

Our findings align with those of previous reports that have highlighted the influence of host genetics in modulating cholestatic responses to viral infections. A similar observation was reported in a case of hepatitis A virus (HAV)‐induced cholestasis [25], a virus that shares important clinical commonalities with HEV [26]. Our findings are further supported by a recent case report by Drexler et al., who described two immunocompetent patients with prolonged intrahepatic cholestasis following acute HEV infection, in the absence of pre‐existing liver disease and of other identifiable causes [11]. Similar to our cohort, both patients exhibited low GGT levels, marked hyperbilirubinemia and histological features consistent with canalicular cholestasis. Next‐generation sequencing revealed heterozygous variants in *ATP8B1* and other hepatobiliary transporter genes, including *ABCB4*, *ABCB11* and *MYO5B*, supporting the hypothesis that host genetic background may modulate the severity of cholestatic responses to HEV. In both studies, the cholestatic phenotype occurred in patients without prior liver disease, reinforcing the concept that HEV may act as a precipitating factor in genetically predisposed but otherwise healthy individuals.

Genetic assessment of patients experiencing prolonged cholestasis following acute viral hepatitis could enhance the personalised infection management in such cases. Targeted interventions, such as the early initiation of drugs that have been shown to interact with the enterohepatic circulation of bile acids such as rifampicin or ileal bile acid transporter (IBAT) inhibitors, could be considered [27].

While the cohort size was limited, our study presents the most extensive series to date of prolonged cholestasis after acute HEV infection, including genetic data. Our experience indicates that the severity of HEV infection may be influenced by host genetics as evidenced by the prolonged cholestatic jaundice in genetically predisposed individuals. These findings underscore the value of integrating genetic analyses to enhance our understanding of the complex pathogenesis, which has also been emphasised in recent guidelines on genetic cholestatic liver disease [28].

## Author Contributions

M.F., and C.J., selected the patients. All authors contributed to the collection of patients' data and analysis. M.F., C.J., and S.K., wrote the manuscript. All authors read and approved the final manuscript.

## Conflicts of Interest

The authors declare no conflicts of interest.

## Data Availability

The data that support the findings of this study are available from the corresponding author upon reasonable request.
